# Supplementary figures and images for: Vitamin D enhances antiviral responses in dengue virus-infected macrophages by modulating early-response gene expression
Source: PLoS One. 2025 Aug 21;20(8):e0330751. doi: 10.1371/journal.pone.0330751 (PMC12370076; doi:10.1371/journal.pone.0330751)

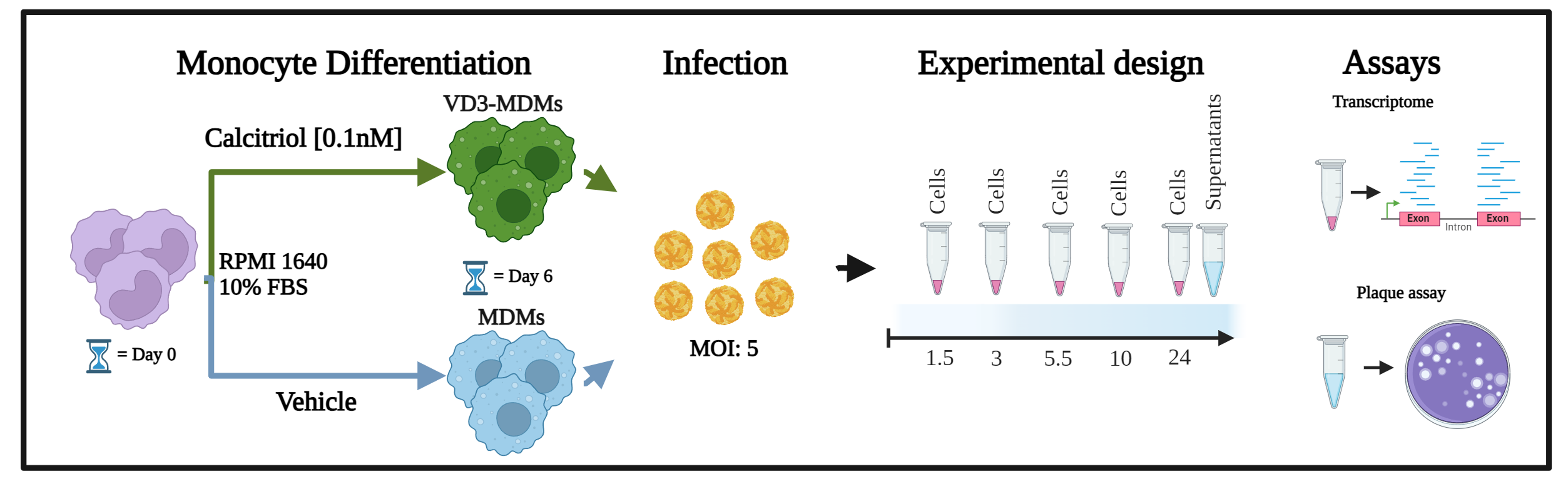

Supplement: S1 Fig — Macrophages were differentiated or not in the presence of calcitriol 0.1 nM. Then, macrophages were infected with DENV-2 at an MOI of 5. Subsequently, cells were collected at 1.5-, 3-, 5.5-, 10-, and 24-h.p.i. Cells were used for bulk RNA sequencing, and supernatants were collected at 24 h.p.i. to quantify viral infectious particles. (TIF) [file pone.0330751.s001.tif]

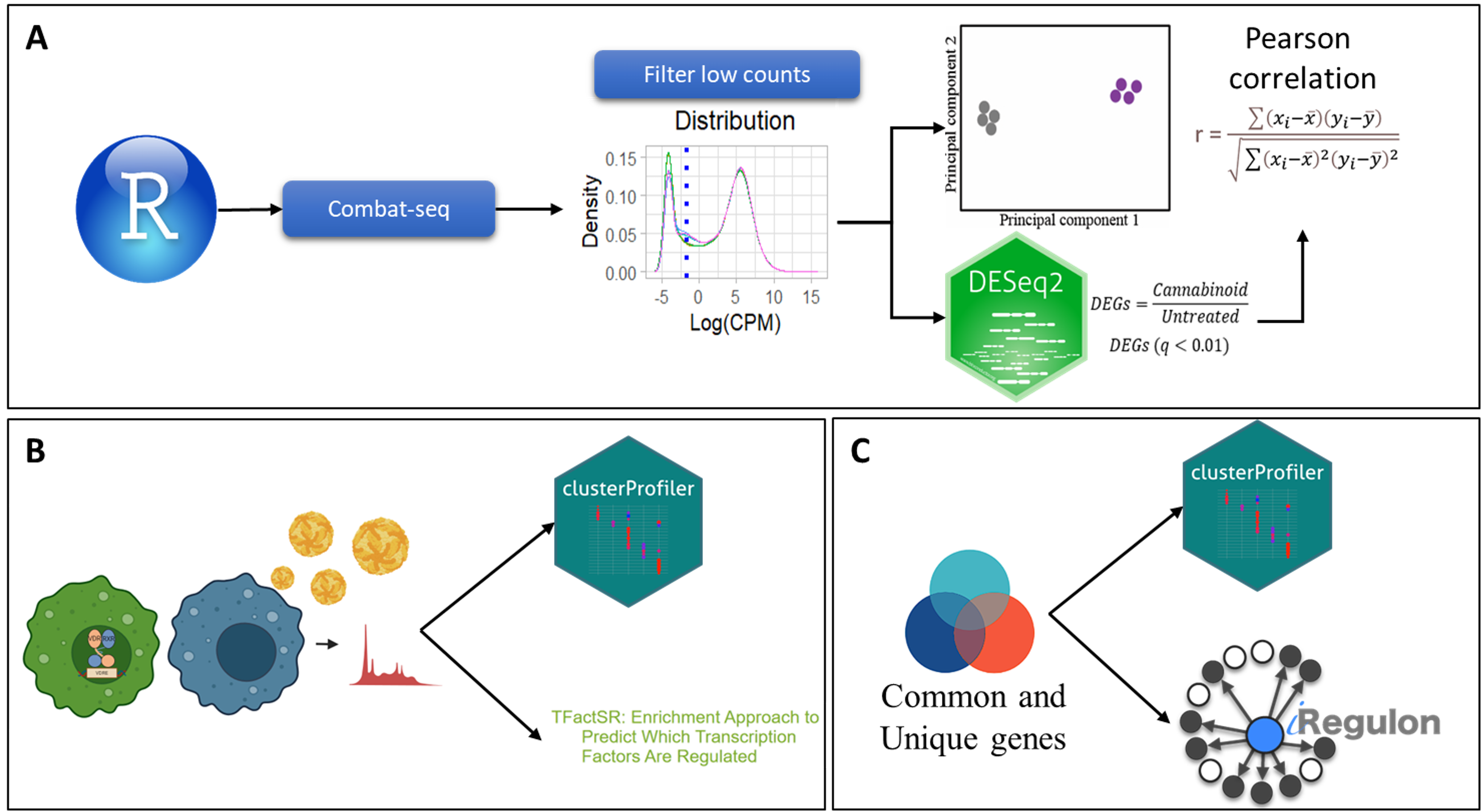

Supplement: S2 Fig — Transcriptome processing and analysis. Raw counts were pre-processed using batch effect correction and low-count filtering. Then, principal component analysis, differential expression analysis, and correlation analysis were performed (A). Gene set enrichment using clusterProfiler and transcription factor enrichment using TFactSR were performed for differentially expressed genes in both macrophage types (B). Unique genes in macrophages were used to search for biological processes using clusterProfiler and transcription factor enrichment by iRegulon. (TIF) [file pone.0330751.s002.tif]

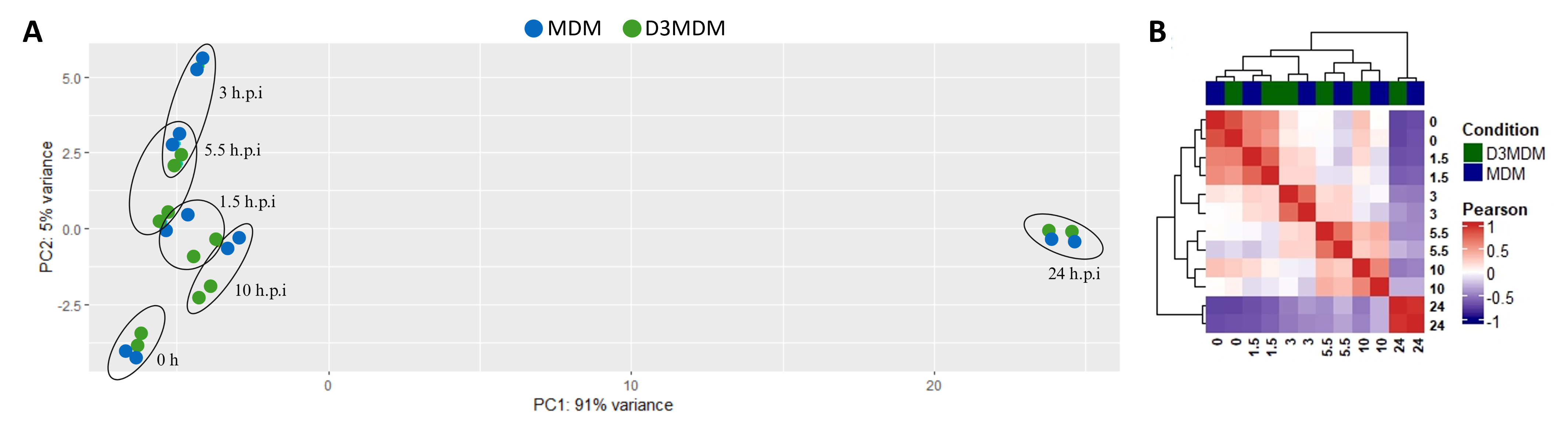

Supplement: S3 Fig — Correlation analysis between samples in DENV-2 infected MDMs (B). (TIF) [file pone.0330751.s003.tif]
