# Supplementary material for: Vitamin D enhances antiviral responses in dengue virus-infected macrophages by modulating early-response gene expression
Source: PLoS One. 2025 Aug 21;20(8):e0330751. doi: 10.1371/journal.pone.0330751 (PMC12370076; doi:10.1371/journal.pone.0330751)
Supplement: Attachment — Submitted filename: Point-by-point_response_to_the_reviewers_auresp_2.docx [file pone.0330751.s006.docx]

Point-by-point response to reviewers

Thank you for your email and the positive feedback on our paper “*Vitamin D Enhances Antiviral Responses in Dengue Virus-Infected Macrophages by Modulating Early-Response Gene Expression*”. PONE-D-25-07206.

Reviewer #2: The authors have made meaningful revisions in response to previous comments, and the manuscript has improved. However, several concerns remain that need to be addressed to strengthen the conclusions:

1. In Figures 3C and 3D, statistical analysis is not indicated. Without clear markers of significance, it is difficult to evaluate the reliability and relevance of the observed differences.

**Answer**: *We thank the reviewer for this observation. We have now included statistical analysis for Figure 3D using a two-way ANOVA model (Expression ~ Treatment * Time), followed by post hoc unpaired t-tests at each time point. However, statistical analysis could not be performed for Figure 3C, as there is only a single value of regulated genes (K) per condition at each time point. Thus, no biological replicates are available for comparison.*

2. The study relies primarily on RNA-seq data and lacks molecular validation. Additional experiments, such as western blotting to assess IRF5-related proteins and viral proteins levels, are needed to support the claim that vitamin D has antiviral activity and could reduce inflammation.

**Answer:** *We thank the reviewer for their insightful comment and suggestions. However, we would like to clarify that the primary objective of this study was to perform a transcriptomic analysis of MDMs differentiated in the presence or absence of vitamin D and subsequently infected with DENV. We aimed to gain a deeper molecular understanding of the functional and antiviral responses modulated by vitamin D in this context.*

3. The manuscript reports an increase in IRF5 expression following vitamin D treatment combined with viral infection. However, the functional link between IRF5 and vitamin D signaling remains unclear. The authors should further explain the relevance of this finding and how it contributes to the proposed antiviral mechanism.

We have added the following lines to address the reviewer’s comment in the Discussion section (lines 499–513):

“These findings suggest that IRF5 functions as a key mediator of the vitamin D-induced antiviral state in human macrophages. By promoting IRF5 expression and its transcriptional activity, vitamin D may prime macrophages to mount a robust early induction of ISGs upon viral infection. This early ISG activation is dependent on IFN-I production and is critical for establishing an antiviral intracellular environment, thereby delaying viral replication and reducing viral antigen levels. Collectively, our findings support the notion that vitamin D orchestrates an antiviral state via the VDR-IRF5-ISG axis, contributing to the observed restriction of DENV replication. Notably, IRF5 has been shown to drive the expression of genes characteristic of M1 macrophage polarization, including TNFα, IL12, and IL-23, and play a critical role in regulating macrophage functional phenotypes [57]. Interestingly, M1 polarized MDM significantly restricts ZIKV replication, an effect associated with the upregulation of ISGs [58].
